# Socio-demographic and clinical features of patients with dementia attended in the psychiatry department

**DOI:** 10.1192/j.eurpsy.2021.1971

**Published:** 2021-08-13

**Authors:** S. Bader, E. Ellouz, R. Abderrahim, M. Abbas, K. Mdhaffer

**Affiliations:** 1 Psychiatry, regional hospital of Gabed, gabes, Tunisia; 2 Neurology, regional hospital of Gabed, gabes, Tunisia

## Abstract

**Introduction:**

Dementia’s prevalence increases due to population aging, it has become a major public health concern.

**Objectives:**

To estimate the incidence of dementia and to describe the socio-demographic and clinical profile of patients attended in the psychiatry department of Gabes (Southern of Tunisia).

**Methods:**

It was a retrospective descriptive study including all the patients who attended for the first time in the psychiatry department of Gabes, from the 1st January, 2010 to December 31, 2018, and who were diagnosed with dementia according to the Diagnostic and Statistical Manual of Mental Disorders (DSM-IV). Socio-demographic and clinical data were assessed. The Mini Mental State Examination (MMSE) was used as a neuropsychological examination.

**Results:**

We included 98 patients. The mean annual hospital incidence of dementia was 2.38%. The mean age was 76.5 ± 9.8 years. Patients were married (68%), illiterate (68%) and jobless (42.9%). A family history of dementia was noted in 39.8% of patients. The common cardiovascular comorbidity was high blood pressure (41.8%). Among our patients, 30 (30.6%) were smokers. The mean age of onset of dementia was 73 ± 11 years. The mean duration of untreated dementia was 3 years [3 months to 11 years]. First symptoms were mainly memory disorders (57.3 %) and behavioral disorders (17.3%) The mean MMSE score was 14 ± 4.8. Alzheimer’s disease was the most frequent etiology of dementia (80 cases, 82.7%).

**Conclusions:**

Our study shows a high incidence of dementia and made it possible to draw up a socio-demographic and clinical profile of dementia patients.

**Disclosure:**

No significant relationships.

